# Structural insights into the interaction of human IgG1 with FcγRI: no direct role of glycans in binding

**DOI:** 10.1107/S1399004715018015

**Published:** 2015-10-31

**Authors:** Vaheh Oganesyan, Yariv Mazor, Chunning Yang, Kimberly E. Cook, Robert M. Woods, Andrew Ferguson, Michael A. Bowen, Tom Martin, Jie Zhu, Herren Wu, William F. Dall’Acqua

**Affiliations:** a Department of Antibody Discovery and Protein Engineering, MedImmune LLC, 1 MedImmune Way, Gaithersburg, MD 20878, USA; b Discovery Sciences, Structure and Biophysics, AstraZeneca Pharmaceuticals, 35 Gatehouse Drive, Mailstop E3, Waltham, MA 02451, USA; c Biopharmaceutical Development, MedImmune LLC, 1 MedImmune Way, Gaithersburg, MD 20878, USA

**Keywords:** CD64, FcγRI, IgG, protein complex, Fc receptor

## Abstract

The three-dimensional structure of a human IgG1 Fc fragment bound to wild-type human FcγRI is reported. The structure of the corresponding complex was solved at a resolution of 2.4 Å using molecular replacement; this is the highest resolution achieved for an unmutated FcγRI molecule. This study highlights the critical structural and functional role played by the second extracellular subdomain of FcγRI. It also explains the long-known major energetic contribution of the Fc ‘LLGG’ motif at positions 234–237, and particularly of Leu235, *via* a ‘lock-and-key’ mechanism. Finally, a previously held belief is corrected and a differing view is offered on the recently proposed direct role of Fc carbohydrates in the corresponding interaction. Structural evidence is provided that such glycan-related effects are strictly indirect.

## Abstract 2

In an effort to identify the critical structural features responsible for the high-affinity interaction of IgG1 Fc with FcγRI, the structure of the corresponding complex was solved at a resolution of 2.4 Å.

## 1. Introduction   

The family of IgG Fc gamma receptors (FcγRs) play a crucial role in controlling the immune response in mammals (Nimmerjahn & Ravetch, 2006 ▸, 2008 ▸; Guilliams *et al.*, 2014 ▸). In humans, this family comprises a complex array of various members (FcγRI, FcγRII, FcγRIIIA and FcγRIIIB) and their allelic variants. These differ in their function (activating *versus* inhibitory), structural features and affinities for different IgG isotypes. FcγRs contain two to three Ig-like C-type domains, a single transmembrane-spanning region (with the exception of FcγRIIIB) and a cytoplasmic tail of varying length. FcγRI, also known as CD64, is the only high-affinity (nanomolar range) receptor and the only one whose extracellular domain (ECD) comprises three individual subdomains (D1, D2 and D3). FcγRI binds IgG1 best, approximately tenfold better than IgG3 and IgG4, and does not bind significantly to IgG2. Mutational studies have previously attributed the high binding affinity of IgG for FcγRI to the second and third subdomains of the receptor (Harrison & Allen, 1998 ▸; Hulett & Hogarth, 1998 ▸). Recently published X-ray crystal structures of human FcγRI bound to IgG1 Fc (Lu *et al.*, 2015 ▸; Kiyoshi *et al.*, 2015 ▸) suggested that D3 does not directly participate in the corresponding interaction. While Lu *et al.* (2015 ▸) reported that FcγRI recognizes Fc glycans and attributed the high affinity between the two partners to this structural feature, Kiyoshi *et al.* (2015 ▸) found that such glycans make only little contribution to the interaction.

We sought to better understand the molecular basis of IgG recognition by FcγRI. For this purpose, we solved the X-ray crystal structure of the complex between the Fc portion of a human IgG1 and unmutated FcγRI at 2.4 Å resolution. Our data allowed a detailed description of the corresponding interface. In particular, we confirm structurally and functionally the critical role played by FcγRI D2. We also explain at a structural level the major energetic contribution of Fc residues spanning positions 234–237 (LLGG). Our study also confirms that the use by Kiyoshi *et al.* (2015 ▸) of an FcγRI molecule mutated at 19 positions did not affect the overall structure and agrees with their findings that glycans do not directly contribute to the interaction.

## 2. Methods   

### 2.1. Host cell-line generation   

An MGAT1 knockout (KO) cell line was generated from Chinese hamster ovary (CHO) K1 cells by knocking out the MGAT1 gene which encodes mannosyl (α-1,3-)-glycoprotein β-1,2-*N*-acetylglucosaminyltransferase. A zinc-finger nuclease pair targeting the coding region of MGAT1 was designed to recognize the gene at the following region: GCCTGCGACCCCCTCACCagccgtGATCCCCATCCTGGTC. ZFN plasmids were transfected into host cells by nucleofection using standard protocols. MGAT1 KO cells were enriched by treatment with phytohemagglutinin (PHA) for two passages. PHA-resistant cells were stained with fluorescent *Galanthus nivalis* lectin (GNA)-FITC to detect high-mannose glycosylation of cell-surface proteins. Strongly staining cells were then subcloned by FACS into 96-well plates. Genomic DNA was isolated from individual wells, amplified using primers flanking the ZFN cut site, denatured, re-annealed and subjected to a CEL1 nuclease assay. CEL1 selectively cleaves re-annealed products that have a mismatch between the two strands. Digested products were run on an agarose gel and amplification products generating a mismatch were further analyzed by DNA sequencing. Clone CATSMGATKO-D4 was identified and contains a frameshift mutation near the ZFN cutting site on both alleles. Recombinant proteins expressed in this cell line exhibit a homogenous Man_5_ glycosylation profile (Shi *et al.*, 2004 ▸).

### 2.2. FcγRI production and purification   

DNA encoding residues 1–277 of human FcγRI ECD was synthesized (Life Technologies, Grand Island, New York, USA) and cloned into a mammalian expression vector under the control of a *Human cytomegalovirus* (CMV) promoter. Briefly, CATSMGATKO-D4 cells were transfected by nucleofection using standard protocols and pools were selected with methionine sulfoximide (MSX; Sigma–Aldrich, St Louis, Missouri, USA). Cell pools were then assessed by flow cytometry for intracellular staining with antihuman FcγRI APC (Life Technologies). The best-expressing pool was expanded and used for the production of secreted FcγRI.

Cells were grown for 13 d, after which the FcγRI-containing medium was collected and passed over a human IgG Sepharose column (GE Healthcare, Piscataway, New Jersey, USA) previously equilibrated with phosphate-buffered saline (PBS) pH 7.2. Following washes to baseline with the same buffer, FcγRI was eluted using Pierce Elution Buffer (Thermo Fisher Scientific, Waltham, Massachusetts, USA). Fractions containing FcγRI were pooled and loaded onto a 5 ml HiTrap SP HP column (GE Healthcare) previously equilibrated with 50 m*M* sodium acetate pH 5.2. Following washes to baseline with the same buffer, FcγRI was eluted in a 0–0.5 *M* NaCl gradient. FcγRI was then dialyzed against 25 m*M* Tris–HCl pH 7.5, 100 m*M* NaCl overnight at 4°C and concentrated to ∼4 mg ml^−1^ using a Vivaspin ultrafiltration device (10 kDa cutoff, Sartorius AG, Bohemia, New York, USA).

### 2.3. Fc production and purification   

DNA encoding a human IgG1 Fc fragment spanning residues 221–446 (EU numbering convention; Kabat *et al.*, 1991 ▸) was cloned into a mammalian expression vector under the control of a *Human cytomegalovirus* (CMV) promoter (Oganesyan *et al.*, 2008 ▸) and transiently transfected into human embryonic kidney (HEK) 293 cells using Lipofectamine (Life Technologies) and standard protocols. Purification was carried out using a HiTrap Protein A column according to the manufacturer’s instructions (GE Healthcare). After overnight dialysis in 25 m*M* Tris–HCl pH 7.5 at 4°C, the protein solution was further applied onto a HiTrap Q HP 5 ml column (GE Healthcare). Following washes to baseline using the same buffer, Fc was eluted in a 0–0.5 *M* NaCl gradient. The protein was then concentrated to ∼10 mg ml^−1^ using a Vivaspin ultrafiltration device (10 kDa cutoff, Sartorius AG). The corresponding SDS–PAGE profile only revealed the presence of one band around 25 or 50 kDa under reducing or nonreducing conditions, respectively (data not shown).

### 2.4. Complex formation and crystallization   

Previously purified FcγRI and Fc were mixed in a 1:1 molar ratio. Further purification of the complex was carried out using a Superdex S200 10/300 GL column (GE Healthcare). The purified complex was then concentrated to ∼5.5 mg ml^−1^ using a Vivaspin concentrator (30 kDa cutoff, Sartorius AG) and subjected to crystallization trials. Sitting-drop crystallization experiments were initially set up in 96-well Intelli-Plates (Art Robbins Instruments, Sunnyvale, California, USA) using a Phoenix crystallization robot (Art Robbins Instruments) and commercially available screens from Hampton Research and Molecular Dimensions. Crystallization optimization was carried out in hanging-drop format using 24-well Linbro plates using varying drop volumes and ratios of protein to reservoir solution in the drop, typically ranging from 1–6 µl and 1:1–5:1(*v*:*v*), respectively. Diffraction-quality crystals were grown from a reservoir solution consisting of 50 m*M* zinc acetate dehydrate, 20% PEG 3350. For cryoprotection, crystals were transferred into the same solution supplemented with 25% glycerol and flash-cooled in liquid nitrogen.

### 2.5. X-ray data collection and structure determination   

A diffraction data set was collected from a single crystal on the IMCA-CAT 17-ID beamline of the Advanced Photon Source (APS) at Argonne National Laboratory (University of Chicago, Chicago, Illinois, USA) equipped with a PILATUS 6M detector (Dectris). 360 diffraction images were recorded at APS using an oscillation range of 0.5°, a crystal-to-detector distance of 401 mm, an exposure time of 1 s and a wavelength of 1.0000 Å. Diffraction data were processed using the *XDS* package (Kabsch, 2010 ▸).

### 2.6. Generation of FcγRI variants   

FcγRI:FcγRIIIA chimeras were designed using the following domain boundaries: FcγRIIIA (D1), 19–106; FcγRIIIA (D2), 107–208; FcγRI (D1), 21–102; FcγRI (D2), 103–187; FcγRI (D3), 188–282. FcγRI and its variants were cloned into an Orip/EBNA-1-based episomal mammalian expression plasmid, pOE (Dimasi *et al.*, 2009 ▸). Proteins were produced by transient transfection of CHO cells in serum-free medium using standard protocols. Cell-culture supernatants were harvested 10 d after transfection and passed through a 0.22 µm sterile filter (PALL Life Sciences, Port Washington, New York, USA). Variants were purified by affinity chromatography using IgG Sepharose 6 Fast Flow (GE Healthcare) and buffer-exchanged into PBS pH 7.2. FcγRIIIA was produced as described by Dall’Acqua *et al.* (2006 ▸). The concentration of the purified proteins was determined from their absorbance at 280 nm.

### 2.7. Binding of FcγRI variants to IgG1   

ELISA plates were coated with FcγRI, FcγRIIIA, individual FcγRI variants or control gp130 at 2 µg ml^−1^ in PBS pH 7.2 at 4°C for 20 h and then blocked with 3%(*v*/*v*) nonfat milk containing 0.1%(*v*/*v*) Tween 20 in PBS pH 7.2 for 1 h at room temperature (RT). A human IgG1 (R347) at concentrations of 10, 5 or 2.5 µg ml^−1^ in PBS pH 7.2 was then added to the wells and incubated for 1 h at RT. HRP-conjugated donkey F(ab′)2 fragment antihuman IgG (H+L) (Jackson ImmunoResearch, West Grove, Pennsylvania, USA) was used as a secondary antibody for 45 min at RT and the plates were developed using tetramethyl­benzidine (TMB; Dako, Carpinteria, California, USA). The signal was quenched with 1 *M* H_2_SO_4_ and read at 450 nm using an EnVision plate reader (PerkinElmer, Waltham, Massachusetts, USA).

## 3. Results and discussion   

### 3.1. Structure of the FcγRI–Fc complex   

**Figure 1:**
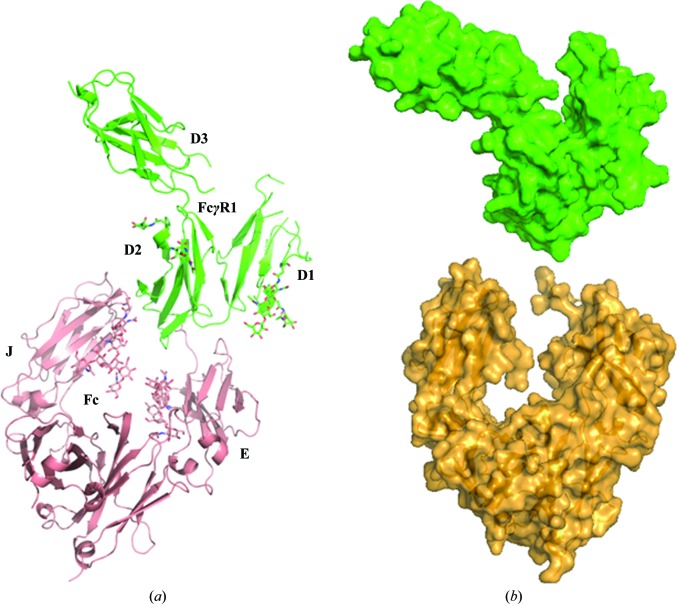
General view of the complex formed between human IgG1 Fc and the ECD of human FcγRI. (*a*) The Fc–FcγRI (salmon/green) complex is shown as ribbons and carbohydrates as sticks. FcγRI D2 appears to be the lone structural contributor to the interface with Fc. (*b*) Surface representation of the Fc–FcγRI complex, in which FcγRI (green) has been moved 20 Å away from Fc (orange) to show the shape complementarity. This and other figures were prepared using *PyMOL* (Schrödinger).

We carried out a crystallographic study of the complex formed between FcγRI ECD and the Fc portion of a human IgG1 in an effort to account for the high affinity of the corresponding interaction. Both proteins were expressed in mammalian cells, purified, complexed and crystallized. The crystals had *C*2 symmetry, with unit-cell parameters *a* = 134.7, *b* = 126.8, *c* = 71.8 Å, β = 118.4°, and diffracted to 2.4 Å resolution. The structure was determined by molecular replacement using *MOLREP* (Vagin & Teplyakov, 2010 ▸). The search model for FcγRI consisted of PDB entry 3rjd (Lu *et al.*, 2011 ▸). The search model for Fc consisted of another Fc portion exhibiting the same amino-acid sequence as in this study, expressed and purified in the same conditions as described above (see §[Sec sec2]2) and the structure of which had been determined at high resolution (1.5 Å; unpublished data). The carbo­hydrate moiety was not included in the search model. One Fc (two identical polypeptides) and one FcγRI molecule were found in the asymmetric part of the unit cell (Fig. 1 ▸ *a*). Iterative refinement/rebuilding of the model was performed with *REFMAC*5 (Murshudov *et al.*, 2011 ▸) and *O* (Jones *et al.*, 1991 ▸). The overall folds of both members of the complex were similar to those of the corresponding templates. Electron density accounted for residues 232–446 of one of the Fc polypeptides (chain E) and for amino acids 236–444 of the other (chain J). Therefore, 11 amino acids at the N-terminus of chain E and 15 amino acids at the N-terminus of chain J, as well as amino acids 445 and 446 at the C-terminus of chain J, were not included in the model. Eight N-terminal and ten C-terminal FcγRI residues, as well as the amino acids corresponding to positions 44–54, 87–90 and 219–222, had no traceable electron density. Both proteins had a number of N-linked carbohydrate chains (Supplementary Fig. S1). Those attached to Fc Asn297 were well visible in the electron density up to the last *N*-acetylglucosamine (GlcNAc). The electron density around FcγRI Asn59, Asn78, Asn152, Asn159 and Asn163 accounted for GlcNAc_2_/Man_1_, GlcNAc_1_, GlcNAc_1_, GlcNAc_1_/Man_1_ and GlcNAc_2_, respectively. Some of those carbohydrates, namely GlcNAc near Asn78 and Man near Asn159, were placed in electron density of much lower quality than others, probably owing to the conformational heterogeneity of sugar moieties (Supplementary Fig. S1). The final refined model contains 5311 protein atoms, 321 carbohydrate atoms, three zinc ions (present in the crystallization solution) and 156 solvent molecules. Data and refinement statistics are shown in Table 1 ▸.

**Figure 2:**
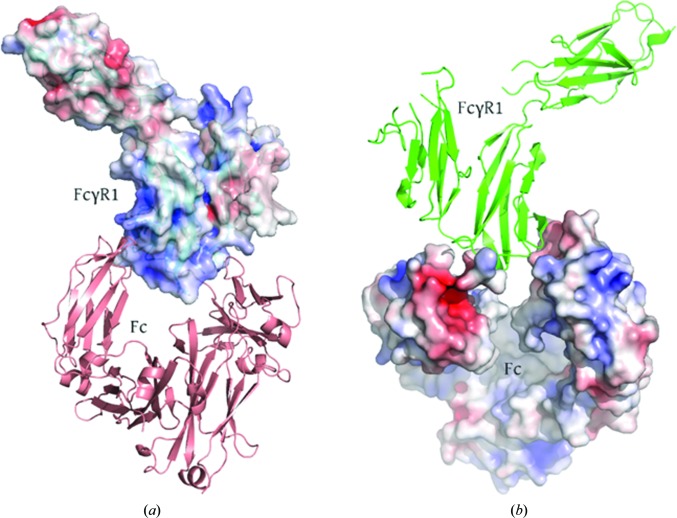
Charge complementarity between Fc and FcγRI. The positive and negative electrostatic potentials are indicated in blue and red, respectively, and were calculated using the *APBS* (*Adaptive Poisson–Boltzmann Solver*; Baker *et al.*, 2001 ▸) plugin in *PyMOL*. Positively charged surfaces on FcγRI (*a*) line up against negatively charged patches of Fc (*b*).

The interface between Fc and FcγRI covers ∼1160 Å^2^ and involves both chains of Fc. No carbohydrate–carbohydrate or protein–carbohydrate inter­actions between Fc and FcγRI were observed. The shape complementarity between Fc and FcγRI is remarkably high (Fig. 1 ▸ *b*). It was estimated at 0.82 using *S*_c_ (0.78 for Fc chain J and 0.84 for Fc chain E; Lawrence & Colman, 1993 ▸), which is higher than the complementarity between the heavy and light chains of antibodies (namely 0.69). The charge complementarity is also remarkable (Figs. 2 ▸ *a* and 2 ▸ *b*). Positively charged FcγRI patches were found facing negatively charged Fc pockets. FcγRI D2 was found to be solely responsible for the interaction with Fc, since no contacts within a distance cutoff up to 5 Å involving D1 and D3 were seen.

**Figure 3:**
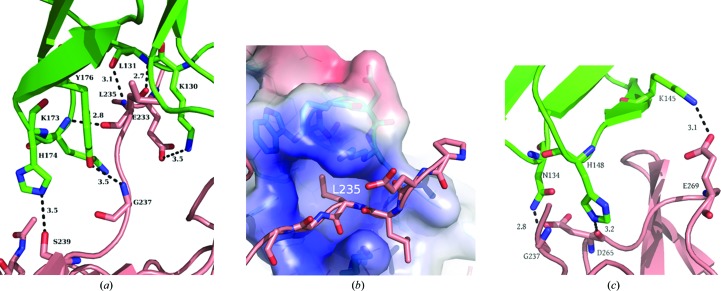
(*a*) Representation of the intermolecular contacts between Fc (E chain, salmon; significance of 0.963) and FcγRI D2 (green). (*b*) FcγRI creates a pocket (‘lock’) to fit Leu235 in the Fc E chain (‘key’; salmon). (*c*) Representation of the intermolecular contacts between Fc (J chain, salmon; significance of 0.348) and FcγRI D2 (green). Fc residues were numbered according to the EU numbering convention (Kabat *et al.*, 1991 ▸). Dotted lines represent hydrogen bonds (distances are given in Å). None of the interfaces involved carbohydrates.

The interfaces between FcγRI and the two Fc chains (E and J) are not equally important. In particular, the interface between FcγRI and Fc chain E constitutes a major interaction area. The complex-formation significance score from *PISA* (Krissinel & Henrick, 2007 ▸) is a remarkable 0.963, and is only slightly lower than that between two Fc polypeptides (1.000). Six hydrogen bonds are formed between FcγRI D2 and the Fc region spanning residues 233-ELLGGPS-239 (Fig. 3 ▸ *a*, Supplementary Table S1). In addition, a very prominent ‘lock-and-key’ feature exists. The ‘key’, corresponding to Leu235 (EL**L**G), is positioned inside an FcγRI pocket exhibiting a positively charged ‘rim’ (Fig. 3 ▸ *b*). The pocket is made of the hydrophobic amino acids Leu105, Trp106, Ala126, Trp127 and Val132 in FcγRI. In addition, these hydrophobic interactions are strengthened by hydrogen bonds formed between the main-chain N and O atoms of Lys173 and Leu131, respectively, in FcγRI with the main-chain O and N atoms of Fc L235 (Fig. 3 ▸ *a*). The other Fc chain (J) established only three hydrogen bonds with FcγRI D2 (Fig. 3 ▸ *c*, Supplementary Table S1).

### 3.2. Comparison of FcγRI–Fc X-ray structures   

While our manuscript was in preparation, two X-ray crystal structures of human FcγRI complexed with IgG1 Fc at resolutions of 3.5 and 1.8 Å were published (Lu *et al.*, 2015 ▸; Kiyoshi *et al.*, 2015 ▸). All three structures are generally similar in terms of domain organization and the relative positions of all polypeptides. Therefore, the FcγRI mutations described by Kiyoshi *et al.* (2015 ▸) do not affect its structure or mode of interaction with Fc. These structures superimpose with r.m.s deviations of 2.2 Å (Lu *et al.*, 2015 ▸) and 0.34 Å (Kiyoshi *et al.*, 2015 ▸) over C^α^ atoms. We also note that the crystal form described here is almost identical to that presented by Kiyoshi *et al.* (2015 ▸) as judged by space group and unit-cell parameters. Lu *et al.* (2015 ▸) proposed that the interaction between FcγRI Arg175 and the Fc carbohydrates can account for the corresponding high affinity. Indeed, the importance and composition of the carbohydrates at Fc Asn297 in FcγR binding is well established. It is believed that glycans allow the Fc C_H_2 domains to maintain a favorable distance and conformation for receptor binding (Radaev *et al.*, 2001 ▸; Jefferis & Lund, 2002 ▸; Arnold *et al.*, 2007 ▸; Feige *et al.*, 2009 ▸). Incremental shortening of IgG1 carbohydrates also results in incremental weakening of the corresponding affinity for FcγR (Mimura *et al.*, 2000 ▸). Structural investigations of such Fcs with truncated carbo­hydrate chains revealed shortening of the overall C_H_2–C_H_2 distance (Krapp *et al.*, 2003 ▸). However, owing to the limited resolution of the structure published by Lu *et al.* (2015 ▸), we find that the glycans were not accurately modeled and exhibited forbidden sugar conformations. Our present structure does not show any glycan-related interaction, which strengthens the previous findings that sugars are not strictly required for complex formation (Sazinsky *et al.*, 2008 ▸) or engaged in significant intermolecular interactions (Kiyoshi *et al.*, 2015 ▸). In particular, the closest distance between the partner molecules in the complex involved the Man_1_ residue attached to FcγRI Asn58 and Fc chain E Ala330, and was estimated at approximately 12 Å.

Furthermore, our structure suggests that Fc Leu235 plays a major role (Fig. 3 ▸ *b*), in agreement with Kiyoshi *et al.* (2015 ▸) and Lu *et al.* (2015 ▸). Our results are also in very good agreement with those of Chappel *et al.* (1991 ▸), who demonstrated the major involvement of this region by introducing the 234-LLGG-237 motif into a human IgG2 and restoring high-affinity binding to FcγRI.

### 3.3. Structural comparison of FcγRI in free and bound states   

**Figure 4:**
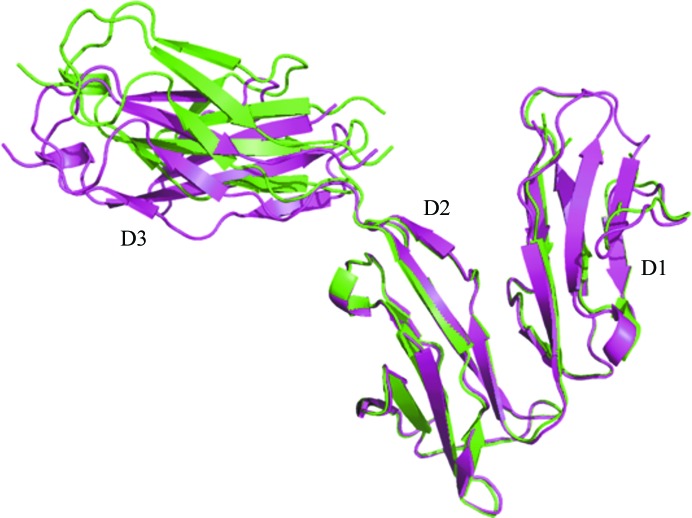
Superimposition of free (PDB entry 3rjd; magenta) and bound (this study; green) FcγRI using D2 C^α^ atoms. No major conformational change can be seen upon Fc binding. However, the position of FcγRI D3, which is not involved in any interactions with Fc, FcγRI D1 or FcγRI D2, appears to depend on both crystal packing and/or the flexibility of the peptide connecting domains 2 and 3. All superimpositions were performed using *LSQKAB* (Kabsch, 1976 ▸).

The X-ray structure of the entire ECD of mammalian cell-derived FcγRI in an unliganded state was previously determined at 2.65 Å resolution by Lu *et al.* (2011 ▸). The three FcγRI subdomains of our structure and this unliganded FcγRI exhibit similar, but not identical, conformations and interdomain interactions. When full-length ECDs were used, the r.m.s deviation over C^α^ atoms was 1.8 Å. When superimposition was carried out at the domain level, the deviations were much smaller and were estimated at 0.38, 0.37 and 0.56 Å for D1, D2 and D3, respectively. We then carried out structural superimposition of both unliganded and bound FcγRI through FcγRI D2, the major contributor of the interaction with Fc, in an effort to detect any change in relative domain orientation upon Fc binding (Fig. 4 ▸). In particular, the acute angle between D1 and D2, previously described in detail by Lu *et al.* (2011 ▸), remained unchanged. We also find that FcγRI D3 is the most misaligned. Since it is not engaged in any contact with either D1 or D2 or with Fc, its position is most likely to be determined by crystal contacts and the flexibility of the connecting peptide between FcγRI D2 and D3. Minor differences in interdomain interactions are shown in Supplementary Table S2.

### 3.4. Relative functional importance of FcγRI domains   

**Figure 5:**
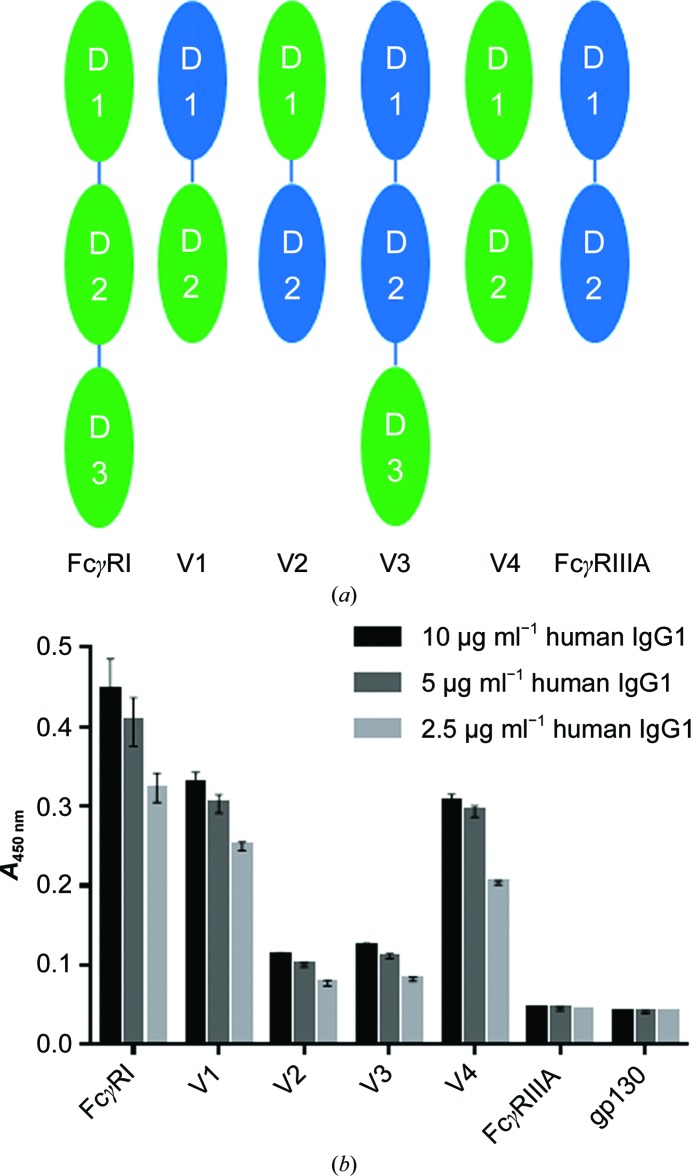
(*a*) Schematic representation of the domain arrangement of human FcγRI, FcγRIIIA and variants thereof. Green and blue ovals correspond to FcγRI and FcγRIIIA domains, respectively. The low-affinity FcγRIIIA/F158 allotype was used to obtain a larger affinity range when characterizing variants. (*b*) Binding of human IgG1 at varying concentrations to FcγR variants as measured by ELISA. Standard deviations are indicated by error bars and represent triplicate measurements within the same experiment.

Our structural analysis suggested that FcγRI D2 is the sole contributor to Fc binding. To investigate whether FcγRI domains I and III play any functional role, we generated several variants in which various domains of FcγRIIIA (F158) and FcγRI were swapped (Fig. 5 ▸ *a*). Binding of a human IgG1 to these variants was then analyzed by ELISA (Fig. 5 ▸ *b*). In the absence of D3 (V4), binding of FcγRI to IgG1 is only slightly weaker when compared with the entire FcγRI ECD, suggesting some minor, probably indirect, role for D3. This is supported by the slight increase in IgG1 binding of FcγRIIIA containing FcγRI D3 (V3) when compared with FcγRIIIA. Variants missing (V1) or including (V4) FcγRI D1 exhibit nearly identical binding to IgG1. This suggests that D1 is interchangeable between FcγRI and FcγRIIIA and does not specifically contribute to IgG1 binding. Importantly, our results highlight the importance of FcγRI D2, as all constructs that lack this domain (V2 and V3) exhibit severely impaired binding to IgG1. Likewise, the V1 construct, in which D2 constitutes the only FcγRI component, binds to IgG1 nearly as well as the entire FcγRI ECD. Taken together, our data show that FcγRI D2 is the most important domain to confer high-affinity binding to Fc. This is in good agreement with previous data using murine molecules (Hulett & Hogarth, 1998 ▸).

## 4. Conclusion   

**Figure 6:**
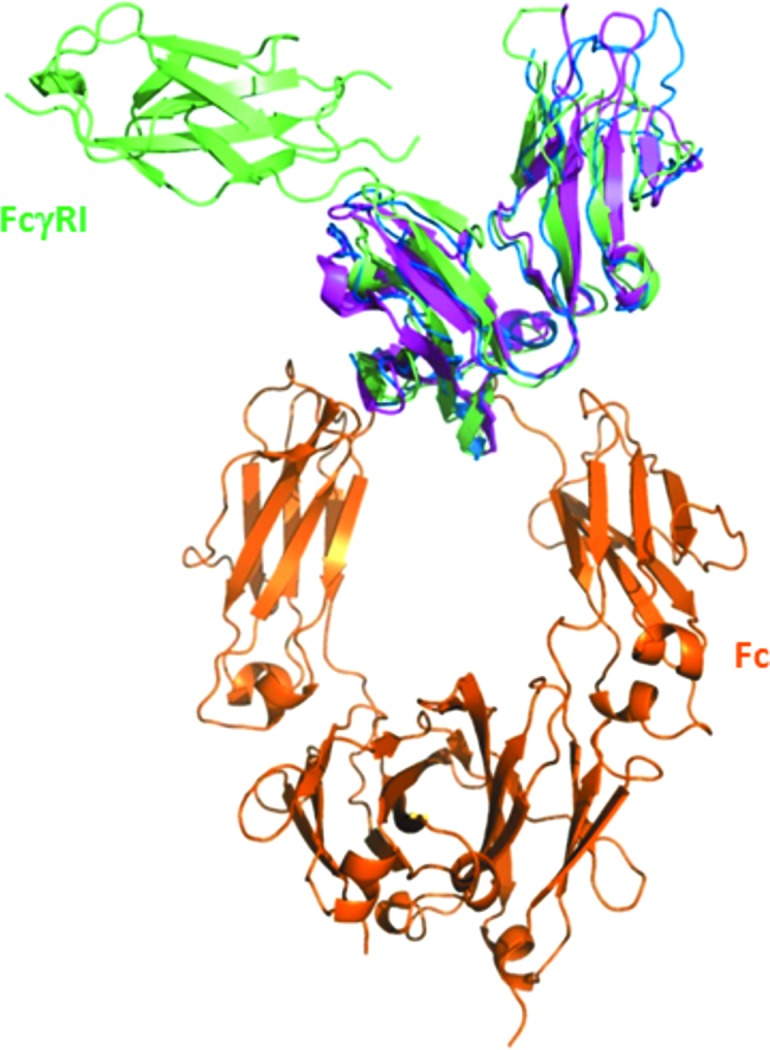
Superimposition of the complex between FcγRI (green) and Fc (orange) with FcγRIIIA–Fc (blue; PDB entry 3sgj) and FcγRIIA–Fc (magenta; PDB entry 3ry6) complexes. The Fc part of the FcγRIIIA–Fc and FcγRIIA–Fc complexes is not shown. All three FcγRs bind in the crevice between Fc C_H_2 domains and superimpose quite well with each other. However, the details of these interactions are quite different as the FcγRIIIA–Fc interface involves carbohydrates from both the receptor and the IgG1. There is no evidence for direct protein–carbohydrate interaction in the FcγRIIA–Fc complex (perhaps owing to the low resolution of the structure).

**Figure 7:**
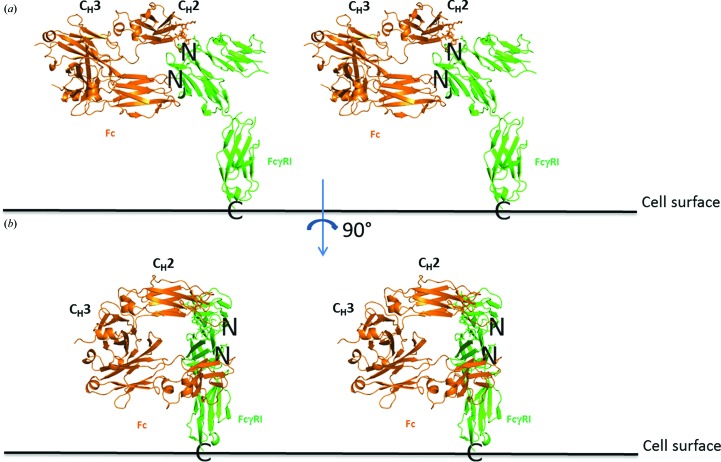
Three-dimensional views of the FcγRI–Fc (green/orange) complex in relation to the presence of Fab arms. (*a*) shows a view in which the Fab arms would be positioned towards the viewer, whereas (*b*) shows a view in which the Fab arms would point towards the right side. The N-terminus of both Fc polypeptides points away from FcγRI, which allows the presence of the corresponding Fab arms.

In an effort to identify the critical structural features responsible for the high-affinity interaction of IgG1 Fc with FcγRI, we solved the structure of the corresponding complex. FcγRI, FcγRII and FcγRIIIA bind to nearly the same place on Fc (Fig. 6 ▸), although the details of these interactions are very different. Unlike the interface of the FcγRIIIA–Fc complex, that between FcγRI and Fc does not contain any carbo­hydrate. It is, however, well known that aglycosylated IgG molecules exhibit very weak to no binding to FcγRI. Therefore, Fc glycans play an important indirect role in this interaction, likely by maintaining a favorable Fc conformation or C_H_2 distance for engaging FcγRI. As a result of the high resolution of our structure, we confirm here that such glycan-related effects are indirect only. The higher resolution also allowed us to provide structural evidence for the important functional role of Fc amino acid Leu235, which is in very good agreement with the studies of Chappel *et al.* (1991 ▸) and Kiyoshi *et al.* (2015 ▸). We have also elucidated the individual role of FcγRI subdomains and find a good agreement between the structural and functional data. In particular, FcγRI D2 constitutes an integral structural and energetic component of the interaction with IgG1 Fc. Finally, our described mode of interaction between FcγRI and Fc is compatible in the context of an interaction with a full-length IgG1 with its Fab arms. Indeed, both N-terminal ends of Fc polypeptides are positioned so as to allow the formation of interchain disulfide bonds and point away from FcγRI (Figs. 7 ▸ *a* and 7 ▸ *b*). Therefore, both Fab arms are expected to have little to no conformational restriction owing to the flexibility of the entire hinge.

## Supplementary Material

PDB reference: IgG1 Fc–FcγRI ECD complex, 4zne

Supporting information file. DOI: 10.1107/S1399004715018015/wa5098sup1.pdf

## Figures and Tables

**Table 1:** X-ray data and model-refinement statistics Values in parentheses are for the highest resolution shell.

Data statistics	
Wavelength ()	1.0000
Resolution ()	86.62.4 (2.432.42)
Space group	*C*2
Unit-cell parameters (, )	*a* = 134.7, *b* = 126.8, *c* = 71.8, = 118.4
Total reflections	134782 (1507)
Unique reflections	39992 (426)
Completeness (%)	98.7 (100.0)
*R*_merge_	0.062 (0.476)
Mean *I*/(*I*)	14.4 (2.4)
Multiplicity	3.4 (3.5)
CC_1/2_	0.995 (0.856)
Refinement statistics	
Resolution ()	86.62.4
*R*_work_	0.201
*R*_free_	0.254
*R*_work+free_	0.203
R.m.s.d., bonds ()	0.011
R.m.s.d., angles ()	1.566
Ramachandran plot†	
Residues in most favored region (%)	93.6
Residues in additionally allowed region (%)	6.4
Residues in generously allowed region (%)	0.0
No. of protein atoms	5311
No. of nonprotein atoms	480
Mean *B* factor (model/Wilson) (^2^)	34.2/43.5

† The Ramachandran plot was produced using *PROCHECK* (Laskowski *et al.*, 1993 ▸).
