# Supplementary material for: Structural insights into the interaction of human IgG1 with FcγRI: no direct role of glycans in binding
Source: Acta Crystallogr D Biol Crystallogr. 2015 Oct 31;71(Pt 11):2354–61. doi: 10.1107/S1399004715018015 (PMC4631484; doi:10.1107/S1399004715018015)
Supplement: Supplementary file 1 [file d-71-02354-sup1.pdf]

BIOLOGICAL CRYSTALLOGRAPHY

Volume 71 (2015)

Supporting information for article:

Structural insights into human IgG1 interaction with FcγRI: no direct role of glycans in binding

Vaheh Oganesyan, Yariv Mazor, Chunning Yang, Kimberly E. Cook, Robert M. Woods, Andrew Ferguson, Michael A. Bowen, Tom Martin, Jie Zhu, Herren Wu and William F. Dall'Acqua

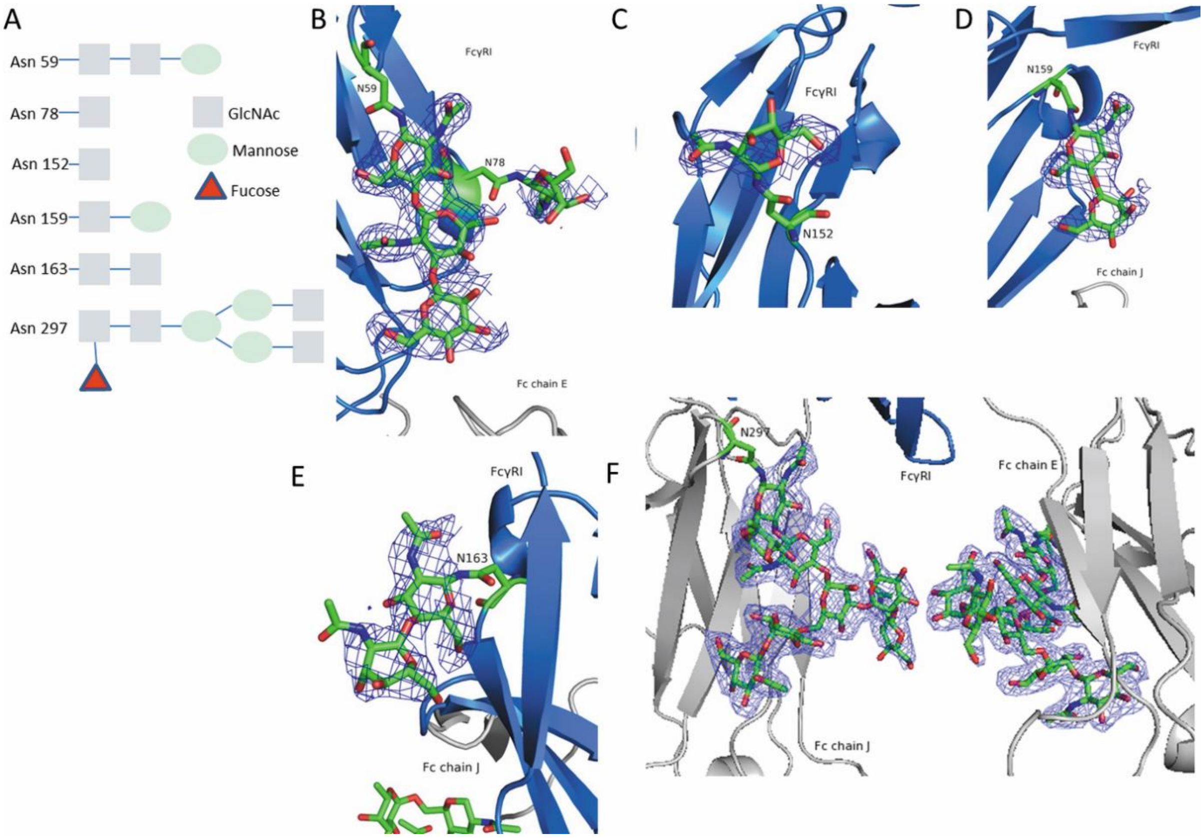

Figure S1 (A) Schematic representation of N-linked oligosaccharides attached to FcγRI and Fc, (B) Omit map showing oligosaccharides attached to FcγRI N59 and N78 in D1. SigmaA weighted $2mF_oDF_c$ electron density map allowed the placement of GlcNAc-GlcNAc-Man near N59; however, the quality of that near N78 was considerably lower and only the first GlcNAc was placed, (C) Omit map showing one oligosaccharide attached to FcγRI N152 in D2. SigmaA weighted $2mF_oDF_c$ electron density map allowed the placement of only one GlcNAc, (D) Omit map showing two oligosaccharides attached to FcγRI N159 in D2. SigmaA weighted $2mF_oDF_c$ electron density map allowed the placement of GlcNAc-Man near N159; however, the quality of that around Man was considerably lower than for GlcNAc, (E) Omit map showing two oligosaccharides attached to FcγRI N163 in D2. SigmaA weighted $2mF_oDF_c$ electron density map allowed the placement of GlcNAc-GlcNAc, (F) Omit map showing oligosaccharides attached to both Fc N297. SigmaA weighted $2mF_oDF_c$ electron density map allowed the placement of bi-antennary glycans. Terminal galactose and sialic acid residues did not show in the electron density. All electron density maps are shown at 0.8σ.

Table S1 Summary of hydrogen bonds and electrostatic interactions formed between FcγRI and Fc.

FcγRI	Distance (Å)	Fc
A:LYS 130[Nζ][a]	3.50	E:GLU 233[Oε2]
A:LEU 131[N]	2.74	E:GLU 233[O]
A:LYS 173[N]	2.85	E:LEU 235[O]
A:HIS 174[Nε2]	3.50	E:SER 239[Oγ]
A:LEU 131[O]	3.07	E:LEU 235[N]
A:TYR 176[Oη]	3.47	E:GLY 237[N]
A:ASN 134[Nδ2]	2.79	J:GLY 237[O]
A:LYS 145[Nζ]	3.07	J:GLU 269[Oε2]
A:HIS 148[Nε2]	3.22	J:ASP 265[O]
A:ARG 102[Nη2]	3.46	E:PRO 329[O]
A:LYS 130[Nζ]	3.50	E:233 GLU[Oε2]
A:HIS 148[Nδ1]	3.30	J:267 SER[Oγ]

[a]Letters in bracket refer to the corresponding interacting atoms.

Table S2 **Hydrogen bonds between FcγR1 D1 and D2 in unliganded and ligand-bound states.**

D1	Hydrogen bond distance (Å) Unliganded	Hydrogen bond distance (Å) Ligand-bound	D2
A:VAL 31[N]	2.97	3.22	A:VAL 109[O]
A:VAL 31[O]	3.09	2.76	A:ARG 112[Nη2]
A:SER 32[O]	2.90	2.63	A:GLN 108[Nε2]
A:GLU 37[Oε2]	2.79	2.70	A:HIS 125[Nε2]
A:GLN 97[O]	2.79	2.68	A:ARG 112[Nη2]
A:GLU 99[Oε1]	3.37	2.95	A:ARG 112[Nη]
